# Supplementary material for: A therapeutic neutralizing antibody targeting receptor binding domain of SARS-CoV-2 spike protein
Source: Nat Commun. 2021 Jan 12;12:288. doi: 10.1038/s41467-020-20602-5 (PMC7803729; doi:10.1038/s41467-020-20602-5)
Supplement: Supplementary file 1 — Supplementary Information [file 41467_2020_20602_MOESM1_ESM.pdf]

**Supplementary information for**

# A Therapeutic Neutralizing Antibody Targeting Receptor Binding Domain of SARS-CoV-2 Spike Protein

Kim et al.

**Supplementary Fig. 1 – 6**

**Supplementary Table 1 – 5**

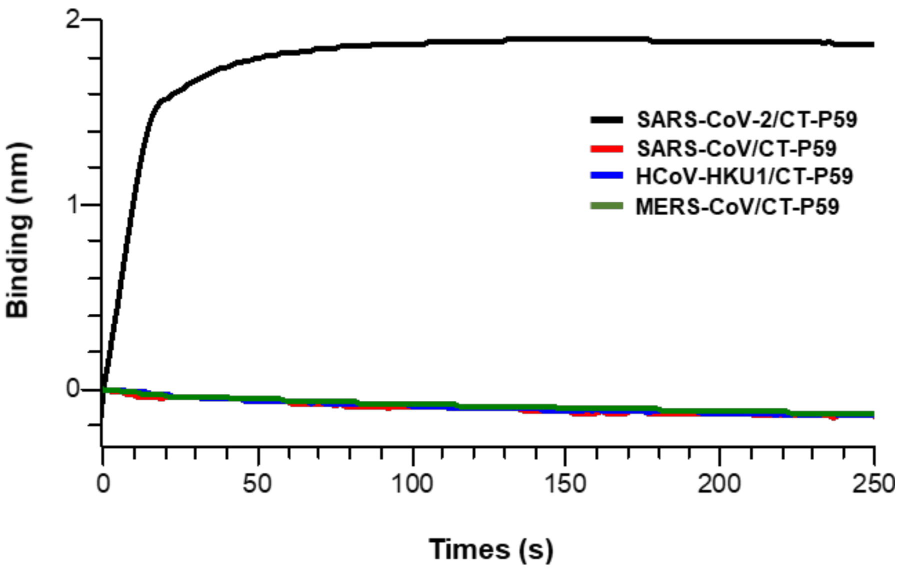


**Supplementary Fig. 1 | Binding specificity of CT-P59 by biolayer interferometry (BLI).** The binding specificity of CT-P59 was measured with four different RBD proteins derived from closely related beta-coronaviruses such as SARS-CoV-2 (black), SARS-CoV (red), HCoV-HKU1 (blue), and MERS-CoV (green) which cause COVID-19, severe acute respiratory syndrome, common cold, and Middle East Respiratory Syndrome, respectively. The binding sensorgrams of CT-P59 toward the four different RBD proteins were presented. Source data are provided as a Source Data file.

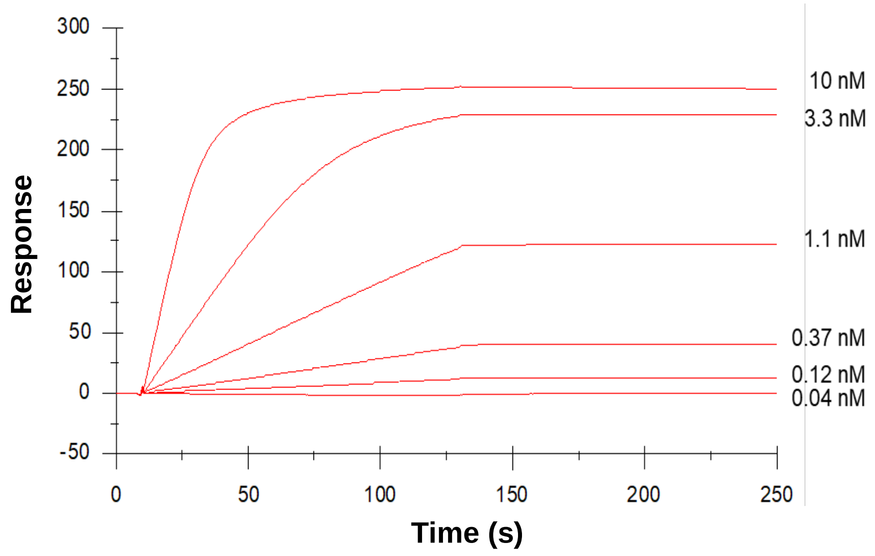


| Sample ID | $K_D$ (M) | ka (1/Ms) | kd (1/s) |
|---|---|---|---|
| CT-P59 | 2.7E-11 | 6.9E+06 | 1.9E-04 |

**Supplementary Fig. 2 | Binding affinity of CT-P59 was determined by SPR.** Several concentrations of CT-P59 were serially injected and dissociated then the dissociation constant was evaluated by sensorgram fitting using the kinetic model (bivalent analyte). Data are expressed as the mean of two independent experiments. ka, binding rate of CT-P59 with SARS-CoV-2 RBD; kd, dissociation rate; $K_D$, affinity of CT-P59 for the RBD. Source data are provided as a Source Data file.

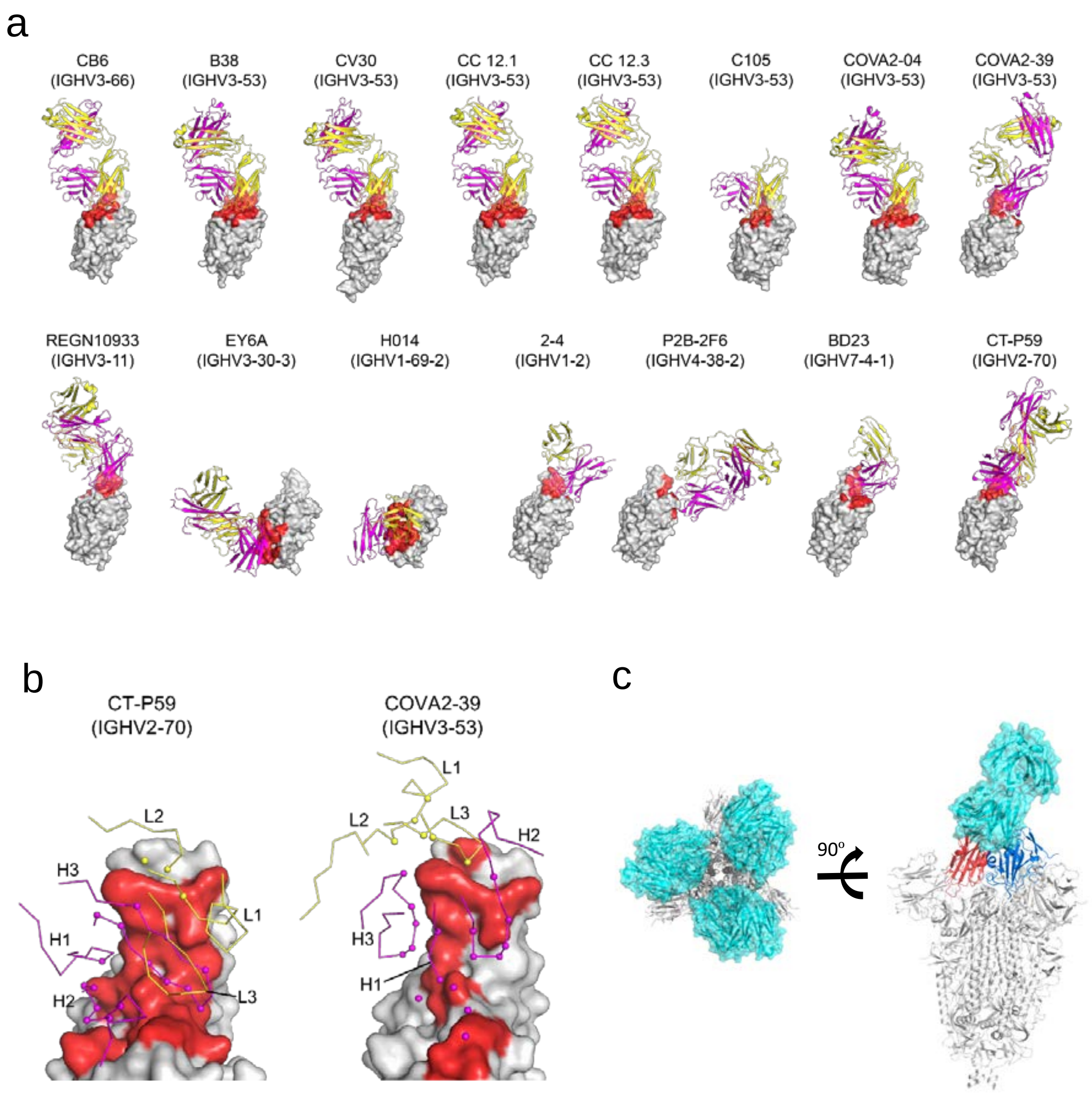


**Supplementary Fig. 3 | Structural comparison with ACE2 blocking neutralizing antibodies with SARS-CoV-2 RBD. a,** Binding orientation between antibodies (cartoon diagram with heavy chain in magenta and light chain in yellow) and SARS-CoV-2 RBD (gray surface model highlighted with epitope region in red). **b,** Epitope comparison between CT-P59 and COVA2-39. Color scheme is same as a. The CDR loops of Fab are shown as a ribbon model, and the RBD interaction residues are indicated by sphere. **c,** Superposition of CT-P59 and "down" conformation of SARS-CoV-2 S protein in trimer (PDB 6VXX). The RBD region interacting with CT-P59 is colored in red and the adjacent RBD is colored in blue

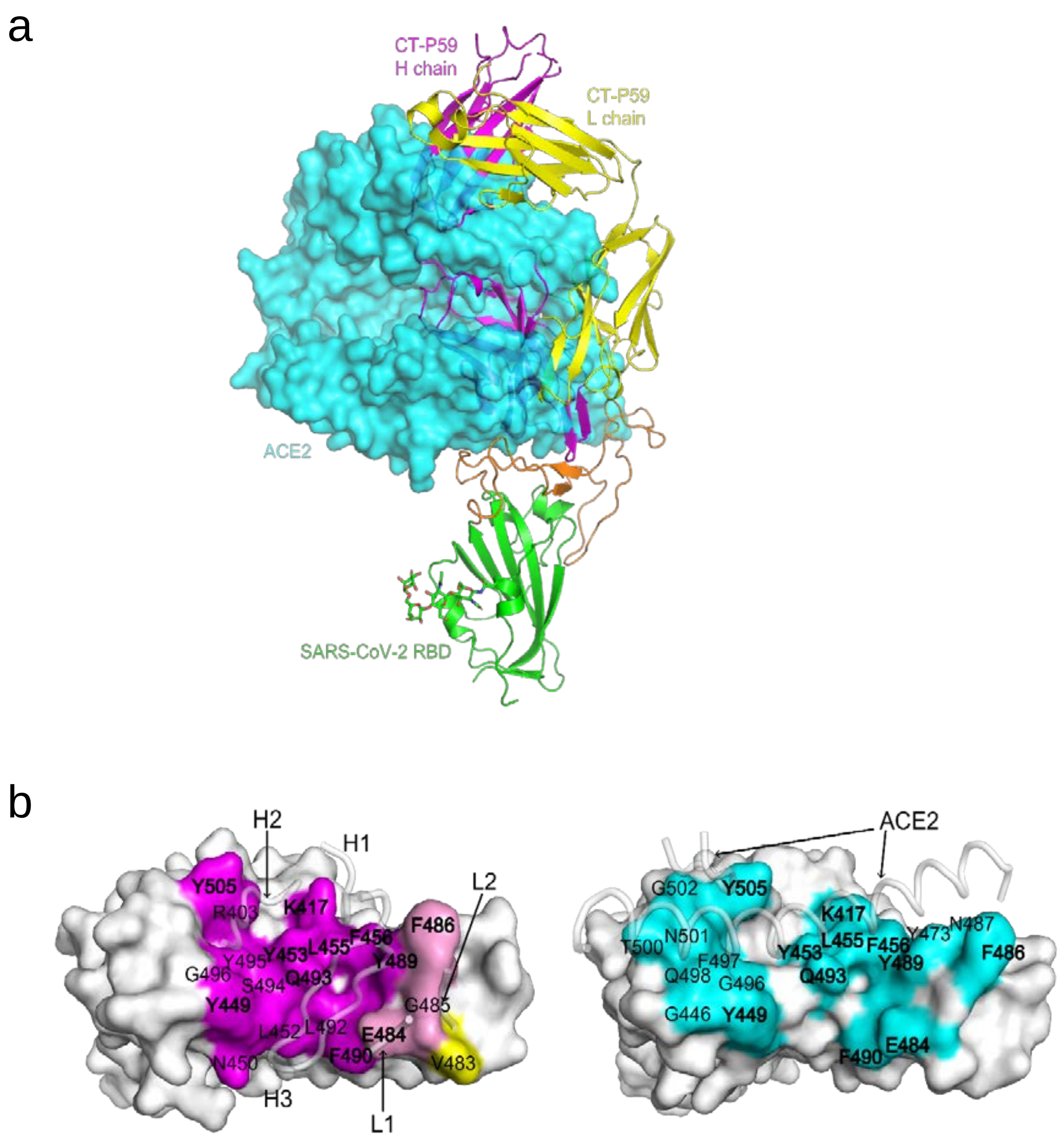


**Supplementary Fig. 4 | SARS-CoV-2 RBD interaction with CT-P59 and ACE2. a,** Superposition of CT-P59 and ACE2. The RBD domains of the CT-P59 Fab/SARS-CoV-2 RBD complex and the RBD-ACE2 complex (PDB 6LZG) were superimposed. ACE2 is shown as a surface model. **b,** Comparison of the RBD interfaces interacting with CT-P59 (left) or ACE2 (right). The residues interacting with heavy chain, light chain or both are coloured in magenta, yellow and pink, respectively. The residues interacting with ACE2 are coloured cyan. The structural segments interacting the RBD are shown in ribbon. The residues interacting both CT-P59 and ACE2 are labelled in bold letters.

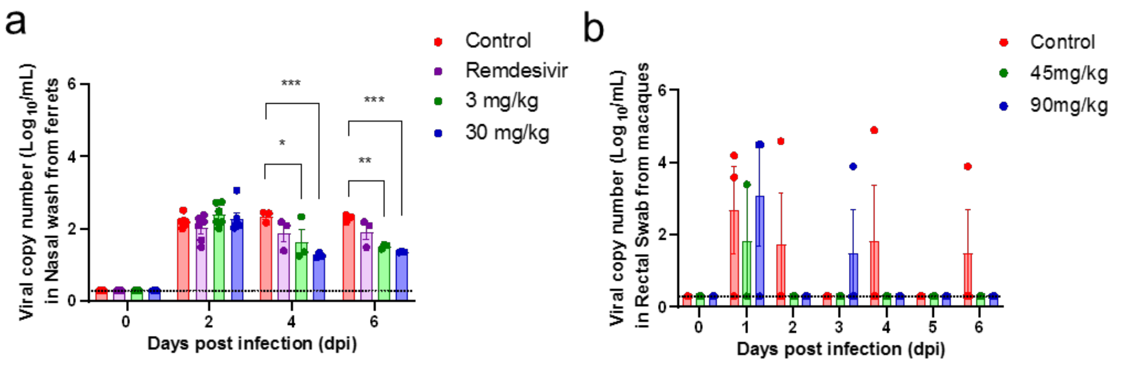


**Supplementary Fig. 5 | Virus quantitation in rectal swabs of animal models.** To investigate the virus titers in gastrointestinal tracts of infected animals, rectal swab specimens were collected at 2, 4 and 6 dpi from ferrets (n=6/group at 0 and 2 dpi; n=3/group at 4 and 6 dpi) and daily in monkeys (three control; two 45 mg/kg; three 90 mg/kg). The viral RNA copy numbers from (**a**) ferrets and (**b**) monkeys were measured by qRT-PCR as described in materials and methods. The RNA copy numbers are shown as means ± SEM below the limit of detection are shown as 0.3 $log_{10}$ viral RNA copies/ml (dashed lines). The asterisks indicate significance between the control and each group as determined by two-way ANOVA and subsequent Dunnett's test. *$P$=0.0032, **$P$=0.0010, ***$P$<0.0001 (**a**).. Source data are provided as a Source Data file.

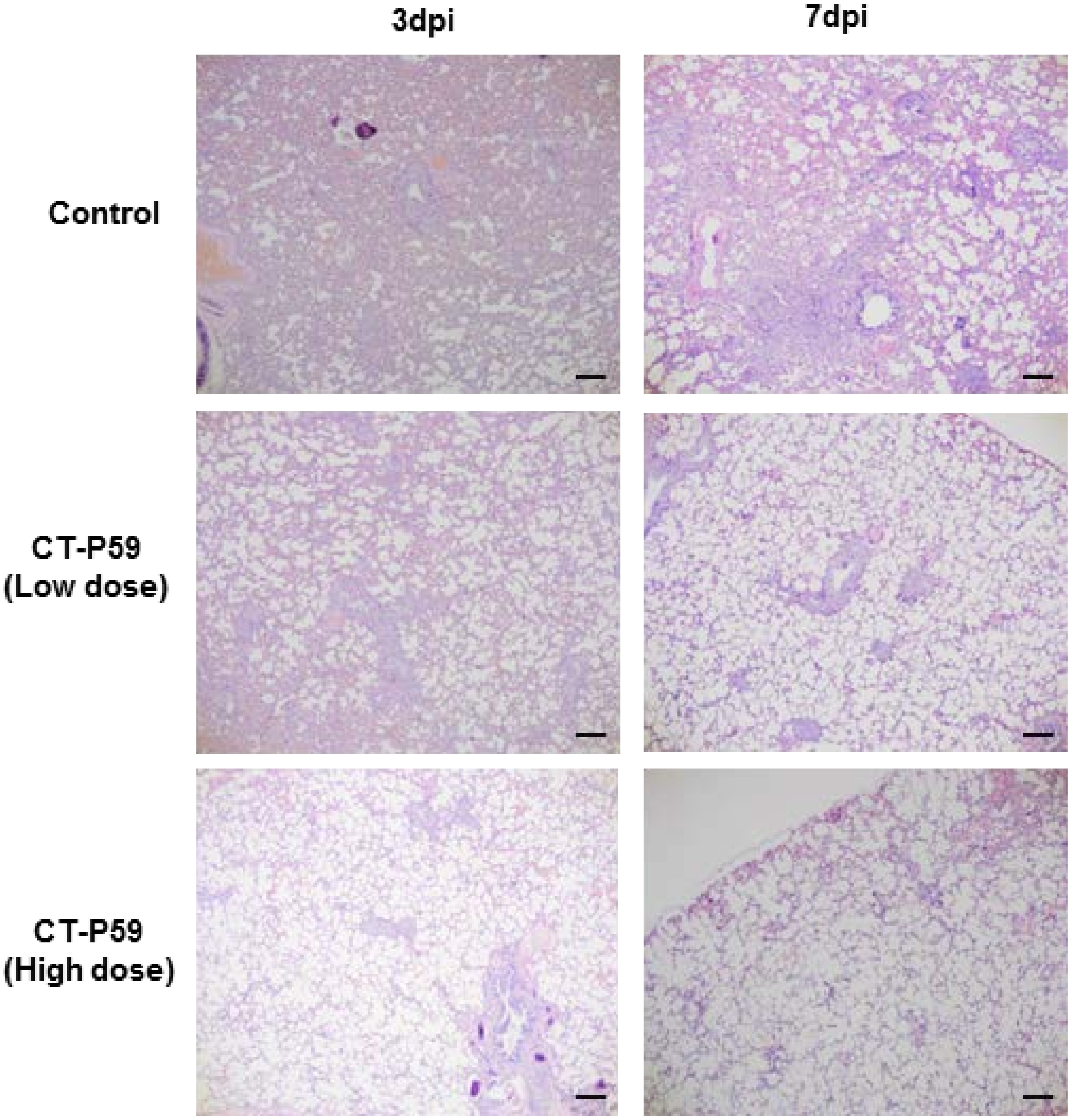


**Supplementary Fig. 6 | H&E-stained sections of animal lung.** Ferrets (n=6/group) were challenged with $10^{5.5}$ $TCID_{50}$/ml of SARS-CoV-2 via, intranasal and intratracheal routes (total 1 ml). Vehicle, 3, or 30 mg/kg of CT-P59 were administered intravenously 24 h after virus inoculation. Three ferrets per group were euthanized at 3 and 7 dpi, and lungs were collected to identify histopathology. For each animal, three sections were evaluated from the same anatomical location. Magnification x40 and Scale bars, 200µm.

**Supplementary Table 1 | Binding affinity between CT-P59 and SARS-CoV-2 RBD mutants determined by BLI**

| Sample ID | $K_D$ (M) | ka (1/Ms) | kd (1/s) |
|---|---|---|---|
| RBD WT | 2.51E-10 | 7.08E+05 | 1.78E-04 |
| RBD A435S | 2.82E-10 | 1.07E+06 | 3.01E-04 |
| RBD F342L | 1.98E-10 | 9.81E+05 | 1.94E-04 |
| RBD G476S | 1.61E-10 | 9.07E+05 | 1.46E-04 |
| RBD K458R | 2.36E-10 | 1.04E+06 | 2.46E-04 |
| RBD N354D | 2.94E-10 | 8.61E+05 | 2.53E-04 |
| RBD V367F | 2.92E-10 | 9.07E+05 | 2.65E-04 |
| RBD V483A | 3.91E-10 | 9.04E+05 | 3.54E-04 |
| RBD W436R | 2.21E-10 | 8.16E+05 | 1.80E-04 |

**Supplementary Table 2 | Data collection and refinement statistics**

| | CT-P59 Fab/SARS-CoV-2-RBD |
|---|---|
| **Data collection** | |
| Space group | I222 |
| Cell dimensions | |
| *a*, *b*, *c* (Å) | 65.6, 167.6, 169.1 |
| α, β, γ (°) | 90, 90, 90 |
| Resolution (Å) | 30-2.71 (2.81 - 2.71) * |
| $R_{sym}$ or $R_{merge}$ | 0.245 (2.145) |
| $I / \sigma I$ | 12.4 (1.6) |
| $R_{sym}$ [a] | 0.245 (2.145) |
| $R_{pim}$ [a] | 0.069 (0.613) |
| $CC_{1/2}$ [b] | 0.997 (0.606) |
| Unique reflections | 25744 (2541) |
| Completeness (%) | 99.8 (99.9) |
| Redundancy | 13.6 (13.1) |
| | |
| **Refinement** | |
| Resolution (Å) | 29.76-2.71 |
| No. reflections | 25734 |
| $R_{work}$ [c] / $R_{free}$ | 0.217 / 0.242 |
| Wilson B-value (Å$^2$) | 58 |
| No. atoms | |
| Protein | 4850 |
| Ligand/ion | 70 |
| Water | 65 |
| *B*-factors | |
| Protein | 65 |
| Ligand/ion | 118 |
| Water | 53 |
| R.m.s. deviations | |
| Bond lengths (Å) | 0.003 |
| Bond angles (°) | 0.588 |

One crystal was used for data collection and refinement.
*Values in parentheses are for highest-resolution shell.

[a] $R_{sym} = \Sigma_{hkl} \Sigma_i | I_{hkl,i} - \langle I_{hkl} \rangle | / \Sigma_{hkl} \Sigma_i I_{hkl,i}$ and $R_{pim} = \Sigma_{hkl} (1/(n-1))^{1/2} \Sigma_i | I_{hkl,i} - \langle I_{hkl} \rangle | / \Sigma_{hkl} \Sigma_i I_{hkl,i}$, where $I_{hkl,i}$ is the scaled intensity of the i$^{th}$ measurement of reflection h, k, l, $\langle I_{hkl} \rangle$ is the average intensity for that reflection, and *n* is the redundancy.

[b] $CC_{1/2}$ = Pearson correlation coefficient between two random half datasets.

[c] $R_{work} = \Sigma ||F_o| - |F_c|| / \Sigma |F_o|$, where $|F_o|$ and $|F_c|$ are the observed and calculated structure factor amplitudes, respectively. $R_{free}$ was calculated with 5% of the data.

**Supplementary Table 3 | Interacting residues between CT-P59 and SARS-CoV-2 RBD**

| SARS-CoV-2 RBD | Heavy chain | SARS-CoV-2 RBD | Light chain |
|---|---|---|---|
| R403 | D56* | V483 | Y33 |
| K417 | S32 | E484 | Y33* |
| Y449 | Y60, R105, R107 | G485 | Y33, D51 |
| N450 | R107* | F486 | Y50, D51* |
| L452 | Y106, R107 | | |
| Y453 | W55, D56* | | |
| L455 | S32, W55, L104 | | |
| F456 | S32, L104 | | |
| E484 | R109*, Y111 | | |
| G485 | Y111 | | |
| F486 | P101, Y111*, Y113 | | |
| Y489 | P101, G102, L104, Y111, | | |
| F490 | Y106, R109 | | |
| L492 | R105,Y106 | | |
| Q493 | D54, W55, N58, L104, R105* | | |
| S494 | R105*,Y106, R107* | | |
| Y495 | N58 | | |
| G496 | N58 | | |
| Y505 | D56, D57 | | |

Van der Waals contact was analyzed at the distance cutoff of 4.5 Å and the H-bonds at the distance cutoff of 3.5 Å (*) using Contact program in CCP4 suite.

**Supplementary Table 4 | Clinical scores of ferrets treated with each candidate antiviral drug**

| Group | Clinical | 0d (n=6) | 1d (n=6) | 2d (n=6) | 3d (n=6) | 4d (n=3) | 5d (n=3) | 6d (n=3) |
|---|---|---|---|---|---|---|---|---|
| Control | **Cough** | 0.00 | 0.00 | 0.50±0.50 | 1.00 | 1.00 | 0.00 | 0.00 |
| | **Rhinorrhea** | 0.00 | 0.00 | 1.00 | 1.00 | 1.00 | 1.00 | 1.00 |
| | **Movement, activity** | 0.00 | 0.00 | 1.33±0.47 | 2.00 | 2.00 | 1.33±0.47 | 1.00 |
| | **Total** | 0.00 | 0.00 | 2.83 | 4.00 | 4.00 | 2.33 | 2.00 |
| CT-P59 (3 mg/kg) | **Cough** | 0.00 | 0.00 | 0.00 | 0.00 | 0.00 | 0.00 | 0.00 |
| | **Rhinorrhea** | 0.00 | 0.00 | 0.67±0.47 | 1.00 | 1.00 | 0.00 | 0.00 |
| | **Movement, activity** | 0.00 | 0.00 | 1.00 | 1.00 | 0.75±0.47 | 0.75±0.47 | 0.00 |
| | **Total** | 0.00 | 0.00 | 1.67 | 2.00 | 1.75 | 0.75 | 0.00 |
| CT-P59 (30 mg/kg) | **Cough** | 0.00 | 0.00 | 0.00 | 0.00 | 0.00 | 0.00 | 0.00 |
| | **Rhinorrhea** | 0.00 | 0.00 | 0.50±0.50 | 1.00 | 1.00 | 0.00 | 0.00 |
| | **Movement, activity** | 0.00 | 0.00 | 1.00 | 1.00 | 0.67±0.47 | 0.67±0.47 | 0.00 |
| | **Total** | 0.00 | 0.00 | 1.50 | 2.00 | 1.67 | 0.67 | 0.00 |
| Remdesivir | **Cough** | 0.00 | 0.00 | 0.00 | 0.67±0.47 | 0.67±0.47 | 0.67±0.47 | 0.00 |
| | **Rhinorrhea** | 0.00 | 0.00 | 0.83±0.37 | 1.00 | 1.00 | 0.00 | 0.00 |
| | **Movement, activity** | 0.00 | 0.00 | 1.00 | 1.33±0.37 | 2.00 | 1.33±0.47 | 1.00 |
| | **Total scores** | 0.00 | 0.00 | 1.83 | 3.00 | 3.67 | 2.00 | 1.00 |

Observational clinical symptoms: Cough, rhinorrhea, movement and activity

- Scores were measured by clinical observation of symptoms for at least 20 minutes in each group of ferrets on the basis of the following criteria:
- Cough: 0 (no evidence), 1 (occasional cough), 2 (frequent cough);
- Rhinorrhea: 0 (no nasal rattling or sneezing), 1 (moderate nasal discharge on external nares), 2 (severe nasal discharge on external nares);
- Movement, activity: 0 (normal movement and activity), 1 (mild reduced movement and activity), 2 (evidence of reduced movement and activity)
- Scoring: Sum the scores of each animal (0, 1, 2) was divided by animal numbers of each group (n) and was summed up each day.

**Supplementary Table 5 | Information of Primers used for SARS-CoV-2 qRT-PCR**

| Name | Amplicon length (bp) | Description | Sequence (5′ to 3′) |
|---|---|---|---|
| S gene | 81 | Forward | ATTCAAGACTCACTTTCTTCCACA |
| | | Reverse | GGTCAACCAAAATGCACAAGCTTTAAACA |
