# Supplementary material for: A therapeutic neutralizing antibody targeting receptor binding domain of SARS-CoV-2 spike protein
Source: Nat Commun. 2021 Jan 12;12:288. doi: 10.1038/s41467-020-20602-5 (PMC7803729; doi:10.1038/s41467-020-20602-5)
Supplement: Supplementary file 3 — Reporting Summary [file 41467_2020_20602_MOESM3_ESM.pdf]

nature research

Corresponding author(s): Young Ki Choi, Soo-Young Lee

Last updated by author(s): Dec 7, 2020

Reporting Summary

Nature Research wishes to improve the reproducibility of the work that we publish. This form provides structure for consistency and transparency in reporting. For further information on Nature Research policies, see our Editorial Policies and the Editorial Policy Checklist.

Statistics

For all statistical analyses, confirm that the following items are present in the figure legend, table legend, main text, or Methods section.

n/a	Confirmed	
☐	☒	The exact sample size (*n*) for each experimental group/condition, given as a discrete number and unit of measurement
☐	☒	A statement on whether measurements were taken from distinct samples or whether the same sample was measured repeatedly
☐	☒	The statistical test(s) used AND whether they are one- or two-sided<br>*Only common tests should be described solely by name; describe more complex techniques in the Methods section.*
☒	☐	A description of all covariates tested
☒	☐	A description of any assumptions or corrections, such as tests of normality and adjustment for multiple comparisons
☐	☒	A full description of the statistical parameters including central tendency (e.g. means) or other basic estimates (e.g. regression coefficient) AND variation (e.g. standard deviation) or associated estimates of uncertainty (e.g. confidence intervals)
☐	☒	For null hypothesis testing, the test statistic (e.g. *F*, *t*, *r*) with confidence intervals, effect sizes, degrees of freedom and *P* value noted<br>*Give P values as exact values whenever suitable.*
☒	☐	For Bayesian analysis, information on the choice of priors and Markov chain Monte Carlo settings
☒	☐	For hierarchical and complex designs, identification of the appropriate level for tests and full reporting of outcomes
☒	☐	Estimates of effect sizes (e.g. Cohen's *d*, Pearson's *r*), indicating how they were calculated

Our web collection on statistics for biologists contains articles on many of the points above.

Software and code

Policy information about availability of computer code

Data collection	SPR data was collected using Biacore T200 control software v3.2.<br>BLI data was collected using Octet Data Acquisition v11.0.<br>X-ray diffraction data was collected using XDS program package (Jan31, 2020).<br>In vitro ADE data was collected using EVOS M7000 v.2.0.1732.0 imaging system.<br>qPCR data were collected by using StepOnePlus™ Real-Time PCR and CFX96 Touch Real-Time PCR Detection System.
Data analysis	PRNT data was analyzed using GraphPad Prism v6.07.<br>SPR data was analyzed using Biacore T200 evaluation software v3.2.<br>BLI data analysis was performed with ForteBio Data Analysis v11.0 and Data Analysis HT v11.0.<br>Model building and refinement were performed using Coot v0.8.9.1 and Phenix v1.18.2-3874. Epitope was identified with CCP4 v7.0.069.<br>All structure figures were generated using Pymol v2.3.0.<br>Viral loads from ferrets, hamsters, and Non-human Primates were analyzed using GraphPad Prism v8.2.0

For manuscripts utilizing custom algorithms or software that are central to the research but not yet described in published literature, software must be made available to editors and reviewers. We strongly encourage code deposition in a community repository (e.g. GitHub). See the Nature Research guidelines for submitting code & software for further information.

Data

Policy information about availability of data

All manuscripts must include a data availability statement. This statement should provide the following information, where applicable:

- Accession codes, unique identifiers, or web links for publicly available datasets
- A list of figures that have associated raw data
- A description of any restrictions on data availability

The data that support the findings of this study are available from the corresponding authors upon reasonable request. The atomic coordinates and structure factor files for the CT-P59 Fab/SARS-CoV-2 RBD complex have been deposited in the Protein Data Bank (PDB) under accession number 7CM4. The publicly available PDB codes used for the structural comparison are 6LZG [http://doi.org/10.2210/pdb6lzg/pdb], 7C01 [http://doi.org/10.2210/pdb7c01/pdb], 7BZ5 [http://doi.org/10.2210/pdb7bz5/pdb], 6XE1 [http://doi.org/10.2210/pdb6xe1/pdb], 6XC3 [http://doi.org/10.2210/pdb6xc3/pdb], 6XC7 [http://doi.org/10.2210/pdb6xc7/pdb], 6XCN [http://doi.org/10.2210/pdb6xcn/pdb], 7JMO [http://doi.org/10.2210/pdb7jmo/pdb], 7JMP [http://doi.org/10.2210/pdb7jmp/pdb], 6XDG [http://doi.org/10.2210/pdb6xdg/pdb], 6ZCZ [http://doi.org/10.2210/pdb6zcz/pdb], 7CAH [http://doi.org/10.2210/pdb7cah/pdb], 6XEY [http://doi.org/10.2210/pdb6xey/pdb], 7BWJ [http://doi.org/10.2210/pdb7bwj/pdb], 7BYR [http://doi.org/10.2210/pdb7byr/pdb] and 6VXX [http://doi.org/10.2210/pdb6vxx/pdb]. Source data are provided with this paper.

Field-specific reporting

Please select the one below that is the best fit for your research. If you are not sure, read the appropriate sections before making your selection.

- [x] Life sciences
- [ ] Behavioural & social sciences
- [ ] Ecological, evolutionary & environmental sciences

For a reference copy of the document with all sections, see nature.com/documents/nr-reporting-summary-flat.pdf

Life sciences study design

All studies must disclose on these points even when the disclosure is negative.

Sample size

Ferrets
No statistical method was used to predetermine sample size. Sample sizes were selected based on previous experience to obtain statistical significance and reproducibility. Animal number (n=6)/group) was also selected to maintain the animals number above n=3/group throughout the study period, considering the interim euthanasia.

golden Syrian hamsters
60 male golden Syrian hamsters were used to statistically analyze in vivo data in this study. Animal number was also selected to maintain the animals number above n=4/group throughout the study period, considering the interim euthanasia

Rhesus monkey
There are three groups investigated, including control group (n=3), low dose group (45mg/kg, n=2), and high dose group (90mg/kg, n=3). Although animals number was below n=3/group in low dose group in accordance with limited animal availability, animal number for control and high dose groups was selected to maintain the animals number above n=3/group throughout the study period.

Data exclusions

Ferrets
No data has been excluded.

golden Syrian hamsters
No exclusions

Rhesus monkey
No data were excluded from the analyses.

Replication

Ferrets
Nasal wash, lung ,and rectal swab: Nasal wash, lung, and rectal swab samples were harvested from all animals at each time point.
Lung histology: For each animal (n=3), three sections were evaluated from the same anatomical location.
Viral titer in lungs: The same anatomical location were evaluated for each animal (n=3).
The evaluation was repeated for some of samples, and principal data could be successfully replicated in duplicate experiments.

golden Syrian hamster
Viral titer in lungs: At 3 and 5 dpi from each group (n=4) were collected for viral load in the lungs.
The evaluation was repeated for some of samples, and principal data could be successfully replicated in duplicate experiments.

Rhesus monkey
Nose swab, throat swab, lung and rectal swab: Nose swab, throat swab, lung and and rectal swab samples were harvested from all animals at each time point.
Viral titer in lungs: At 6 dpi from each group (Control: n=3, 45 mg/kg: n=2 and 90 mg/kg: n=3) were collected for viral load in the lungs.
As for the viral titers experiments, the animals for each group (at each detecting time-point) were tested, and some of

	them were repeated twice separately by two investigators. Principal data could be successfully replicated in duplicate experiments.
Randomization	For all animal studies, ferrets were randomly assigned to each group.<br>Hamsters were randomly segregated into 5 groups.<br>For the monkey study, the animals were randomly divided in to the three groups before the infection.<br>For in vitro studies, all samples, e.g. cells for PRNT, recombinant proteins for BLI, were allocated randomly into experimental groups.
Blinding	The investigators were not blinded to group allocation during the collection of specimens from animal (ferrets and hamster), but were blinded to data analysis. During the study, all monkeys were coded. The technicians did not know which samples are from control or antibody-treated group, with or without virus infection.<br>Blinding was not relevant to ferrets and hamsters studies, since animals were not previously coded. Although blinding was not kept during randomization, any bias was not applied for randomization, and data acquisition and analysis were performed blinded.

Reporting for specific materials, systems and methods

We require information from authors about some types of materials, experimental systems and methods used in many studies. Here, indicate whether each material, system or method listed is relevant to your study. If you are not sure if a list item applies to your research, read the appropriate section before selecting a response.

Materials & experimental systems

n/a	Involved in the study
☐	☒ Antibodies
☐	☒ Eukaryotic cell lines
☒	☐ Palaeontology and archaeology
☐	☒ Animals and other organisms
☐	☒ Human research participants
☒	☐ Clinical data
☒	☐ Dual use research of concern

Methods

n/a	Involved in the study
☒	☐ ChIP-seq
☒	☐ Flow cytometry
☒	☐ MRI-based neuroimaging

Antibodies

Antibodies used	Mouse Anti-nucleocapsid antibody: Sino Biological, Cat. No. 40143-MM05;<br>HRP-conjugated anti-mouse IgG antibody: Southern Biotech, Cat. No. 1030-05.<br>No antibody was used for this study except two antibodies described here.<br>Anti-Human IgG is directly conjugated antibody onto commercially available biosensor which was used for evaluation of the binding affinity of CT-P59 to SARS-CoV-2 RBD in this study.
Validation	For Mouse Anti-nucleocapsid antibody:<br>https://www.sinobiological.com/antibodies/cov-nucleocapsid-40143-mm05;<br>For HRP-conjugated anti-mouse IgG antibody:<br>https://www.southernbiotech.com/?catno=1030-05&type=Polyclonal#&panel1-1&panel2-1;<br>For biosensor to capture human IgG Fc:<br>https://www.sartorius.com/en/products/protein-analysis/biosensors-and-kits/anti-higg-fc-capture-ahc-biosensors.

Eukaryotic cell lines

Policy information about cell lines

Cell line source(s)	VeroE6 cells: ATCC, CRL-1586;<br>Vero cells: ATCC, CCL-81;<br>Raji cells: ATCC, CCL-86;<br>U937 cells: ATCC, CRL-1593.2.
Authentication	The cell lines were not authenticated since they were purchased commercially and not commonly misidentified.
Mycoplasma contamination	The cells were tested for mycoplasma contamination. No mycoplasma contamination was observed.
Commonly misidentified lines (See ICLAC register)	The cell lines used in this study do not appear on the ICLAC register.

Animals and other organisms

Policy information about studies involving animals; ARRIVE guidelines recommended for reporting animal research

Laboratory animals	Fourteen to eighteen months-old female ferrets (6/group, n=18) were used for SARS-CoV-2 infections.<br>Hamster: six-week-old male golden Syrian hamster.<br>Eight 5- to 7-year-old rhesus monkeys (Macaca mulatta, 5 males, 3 females).
Wild animals	The study did not involve wild animals.
Field-collected samples	The study did not involve samples collected from the field.
Ethics oversight	The ferret experiment protocols was approved by the Medical Research Institute, a member of Laboratory Animal Research Center of Chungbuk National University (LARC) (approval number: CBNUA-1352-20-02), and conducted in BSL3 facility (KCDC-14-3-07).<br>The Animal Care and Use Committee at the Agency for Defense Development approved the hamster experiments (ADD-IACUC-20-12).<br>For the monkey study, all procedures were performed in a Biosafety Cabinet class Ⅱ in the ABL-3 facility in the KNPRC at the KRIBB (permit no. KRIBB-AEC-20168).

Note that full information on the approval of the study protocol must also be provided in the manuscript.

Human research participants

Policy information about studies involving human research participants

Population characteristics	We used the blood from one convalescent patient with COVID-19 (male, 84 years old) in South Korea.
Recruitment	A patient was randomly selected with no selection criteria for this study among convalescent patients who agreed to provide blood. The convalescent patient agreed to provide the biospecimen for further diagnostic and scientific research. All specimens were fully anonymized.
Ethics oversight	The research protocol was approved by Seoul National University Hospital Institutional Review Board (IRB No. 2002-105-110). The full name of the board/committee has been described in the manuscript as provided here.

Note that full information on the approval of the study protocol must also be provided in the manuscript.
